# Molecular Mechanisms of Resistance to FLT3 Inhibitors in Acute Myeloid Leukemia: Ongoing Challenges and Future Treatments

**DOI:** 10.3390/cells9112493

**Published:** 2020-11-17

**Authors:** Sebastian Scholl, Maximilian Fleischmann, Ulf Schnetzke, Florian H. Heidel

**Affiliations:** 1 Klinik für Innere Medizin II, Abteilung Hämatologie und Internistische Onkologie, Universitätsklinikum Jena, Am Klinikum 1, 07740 Jena, Germany; maximilian.fleischmann@med.uni-jena.de (M.F.); ulf.schnetzke@med.uni-jena.de (U.S.); 2 Innere Medizin C, Universitätsmedizin Greifswald, Sauerbruchstrasse, 17475 Greifswald, Germany; florian.heidel@uni-greifswald.de

**Keywords:** acute myeloid leukemia, AML, FMS-like tyrosine kinase 3, FLT3, FLT3-ITD, FLT3-TKD, resistance, midostaurin, quizartinib, gilteritinib, crenolanib

## Abstract

Treatment of FMS-like tyrosine kinase 3 (FLT3)-internal tandem duplication (ITD)-positive acute myeloid leukemia (AML) remains a challenge despite the development of novel FLT3-directed tyrosine kinase inhibitors (TKI); the relapse rate is still high even after allogeneic stem cell transplantation. In the era of next-generation FLT3-inhibitors, such as midostaurin and gilteritinib, we still observe primary and secondary resistance to TKI both in monotherapy and in combination with chemotherapy. Moreover, remissions are frequently short-lived even in the presence of continuous treatment with next-generation FLT3 inhibitors. In this comprehensive review, we focus on molecular mechanisms underlying the development of resistance to relevant FLT3 inhibitors and elucidate how this knowledge might help to develop new concepts for improving the response to FLT3-inhibitors and reducing the development of resistance in AML. Tailored treatment approaches that address additional molecular targets beyond FLT3 could overcome resistance and facilitate molecular responses in AML.

## 1. Introduction

Activating FMS-like tyrosine kinase 3 (FLT3) mutations are detected in about one-third of patients with acute myeloid leukemia (AML) at diagnosis. The majority of these mutations are FLT3-internal tandem duplications (ITD) [1,2]. Approximately 25% of patients with AML are diagnosed as FLT3-ITD-mutated. The prognostic impact of FLT3-ITD depends on the allelic ratio (ITD/wildtype allele) and co-occurrence of a nucleophosmin-1 (NPM1) mutation. However, the European Leukemia Net (ELN) and National Comprehensive Cancer Network (NCCN) guidelines use different approaches for prognostic stratification of newly diagnosed patients with AML and FLT3-ITD mutations [3,4]. In general, the presence of co-occurring mutations has substantial effects on prognosis in AML [5].

Besides activating FLT3-ITD mutations, FLT3 tyrosine kinase domain (TKD) mutations are detected in 7–11% of patients with AML [6,7]. TKD mutations predominantly occur at codons D835 or I836 and can be associated with primary resistance to FLT3 inhibitors [8,9]. In contrast to the impact of FLT3-ITD, data concerning the prognostic significance of FLT3-TKD mutations controversially discussed and depend on the co-occurrence of other mutations, e.g., FLT3-TKD and NPM1 mutations [10,11]. FLT3-ITD mutations can be associated with adverse prognosis, particularly, in patients with a high allelic ratio of ≥0.5. Furthermore, FLT3-ITD mutations increase the relapse risk following intensive induction chemotherapy, although most clinical trials failed to demonstrate impact of FLT3-ITD mutations on complete remission rates following induction therapy.

Patients with FLT3-ITD-positive AML have shown a higher probability of relapse following conventional chemotherapy and allogeneic stem cell transplantation (ASCT) [12]. Although the prognostic benefit of ASCT for FLT3-ITD-positive AML is widely accepted, deeper insight regarding the underlying molecular mechanisms are clearly warranted. Aspects concerning depth of remission before ASCT are currently being evaluated in ongoing clinical trials with 2nd generation FLT3 inhibitors such as quizartinib or gilteritinib [13,14].

## 2. FLT3-ITD Signaling Pathways and Diversity of ITD Mutations

FLT3-ITD and FLT3-TKD mutations lead to constitutive activation of multiple downstream signaling pathways and this results in increased proliferation, reduced susceptibility to apoptosis, and inhibition of myeloid differentiation [15,16]. Importantly, activation patterns of downstream signaling are dependent on the presence of either FLT3-ITD or FLT3-TKD mutations and result in distinct AML phenotypes [17,18]. ITD-mediated FLT3-activation is caused by destabilization of its autoinhibitory juxtamembrane (JM) domain and results in constitutive activation of STAT, MAPK-ERK, and PI3K signaling [19,20]. Phosphorylation of STAT family members is an essential step that depends on activation of SRC kinase. Of note, this signaling pathway is not observed downstream of FLT3-TKD mutations or ligand-activated FLT3-wildtype receptors. STAT5 activation has pleiotropic effects on cellular transformation. This includes the activation of the serine/threonine kinase PIM-1, which is responsible for stabilization of the 130 kDa FLT3-ITD variant and accelerates STAT5 activation [21]. Furthermore, PIM-1 can increase survival or mediate resistance to FLT3 inhibitors by activating the anti-apoptotic protein MCL-1 [22,23]. Interestingly, activation of MCL-1 is also a downstream effect of STAT5 signaling that contributes to the maintenance of FLT3-ITD-positive leukemic stem cells [24].

Impaired myeloid differentiation in FLT3-ITD-positive AML blasts is a consequence of deregulated gene expression and is induced by multiple signaling pathways. FLT3-ITD mutations lead to downregulation of myeloid transcription factors, such as PU.1 or CEBPA [25]; the latter has been shown to be phosphorylated and inhibited by FLT3-ITD-signaling [26]. Additionally, RGS2, an important regulator of myeloid differentiation, is repressed by FLT3-ITD [27]. In contrast, high expression of RUNX1 has been identified in FLT3-ITD-positive AML cells, which may contribute to the development of AML and blockade of differentiation in FLT3-ITD blasts [28]. Thus, profound changes in transcriptional programs may block differentiation in the presence of FLT3-ITD mutations.

Maturation of FLT3-ITD receptor represents a complex process of post-translational modifications that includes multiple steps of glycosylation occurring in the Golgi apparatus (GA) and endoplasmic reticulum (ER). Importantly, the majority of FLT3-ITD molecules can be detected in the ER of AML cells, while a relatively small amount of FLT3-ITD protein is located at the cell membrane [29]. The hypoglycosylated 130 kDa species of FLT3-ITD that is retained in the ER can induce PIM-1 expression via aberrant STAT5 signaling [30]. Importantly, critical differences in downstream signaling are observed depending on the cellular localization of FLT3-ITD [30]. Surface localization of FLT3-ITD primarily leads to constitutive activation of PI3K/AKT and MEK-ERK signaling, while FLT3-ITD bound to the GA or ER predominantly activates the STAT5/PIM pathway. Notably, inhibition of FLT3-ITD glycosylation can lead to further reduction in surface FLT3-ITD and consecutive decrease of downstream signaling. Protein stability of oncogenic kinases, such as FLT3-ITD, is highly dependent on chaperones, including heat shock proteins (e.g., HSP90) [31]. Inhibition of these chaperones may lead to induction of apoptosis through enhanced proteasomal degradation of mutant FLT3 [32].

The diversity of FLT3-ITD mutations in regard to their localization and length indicates that ITD mutations are in most cases unique for each AML patient [33]. This sequence diversity increases the complexity of FLT3-ITD biology regarding its use as a molecular marker for monitoring the minimal residual disease [34]. Notably, only 70% of FLT3-ITD mutations are typically localized within the JM region of FLT3. The remaining 30% of FLT3-ITD insertions can be detected in TKD1 and are associated with a different prognostic impact than JM-ITDs [35,36]. The upstream localization of FLT3-ITD mutations correlates with a higher rate of complete remission following induction chemotherapy of patients with FLT3-ITD-positive AML [37]. Nevertheless, the molecular mechanisms that lead to differential signaling depending on ITD localization and size are not fully understood.

## 3. FLT3 Inhibitors

Several FLT3 inhibitors have been developed for treatment of FLT3-mutated AML. However, only two (midostaurin and gilteritinib) are currently approved by the Food and Drug Administration (FDA) and the European Medical Agency (EMA) for distinct clinical indications. More promising compounds have been investigated so far in clinical trials, and comprehensive data exist to understand the molecular mechanisms contributing to FLT3-inhibitor resistance. Table 1 summarizes the key features of FLT3 inhibitors that are either approved or under investigation in advanced phases of clinical trials. These inhibitors can be divided into two distinct functional subtypes based on their mechanism of action. While type I inhibitors can bind and inhibit both active and inactive states of the mutated receptor, type II inhibitors are restricted to binding inactive receptor molecules. This results in distinct patterns of inhibition. The second-generation FLT3 inhibitor quizartinib (type II) cannot inhibit FLT3-TKD mutations at therapeutically relevant doses, which may result in the development of secondary resistance. Importantly, the in vitro sensitivity of FLT3-TKD mutations towards type II FLT3 inhibitors can significantly differ depending on the specific amino acid substitution within the D835 codon. In contrast, the type I FLT3 inhibitors gilteritinib or crenolanib can inhibit both FLT3-ITD and FLT3-TKD and therefore show less development of resistance [38,39]. Furthermore, first-generation inhibitors are less specific for FLT3 and show significant “off target” effects. Conversely, second-generation inhibitors are more specific and characterized by a narrow kinome-profile. Below, we provide a detailed description of FLT3 inhibitors in clinical use:

Sorafenib is a type II inhibitor that has been approved for the treatment of several solid tumors. It is one of the most intensively evaluated compounds in AML with activating FLT3 mutations. Sorafenib inhibits specifically FLT3-ITD. FLT3-TKD mutations are not inhibited at therapeutic concentrations. Besides diarrhea and fatigue, skin toxicity (e.g., hand–foot syndrome) is frequently observed and may eventually lead to treatment discontinuation [40]. Despite promising results in several clinical trials (including reports on survival benefit after ASCT), sorafenib has not been approved by the FDA or EMA [41] so far.

Midostaurin is a potent type I multikinase inhibitor and targets constitutively activated FLT3 receptor. Midostaurin has been approved in combination with induction and consolidation chemotherapy as well as for maintenance therapy of AML patients who do not undergo ASCT. As shown in the randomized phase 3 RATIFY trial, most relevant side effects of midostaurin study include pulmonary complications such as drug-induced pneumonitis. Frequent side effects also include nausea, vomiting, edema, bruising, and QTcF prolongation [12,42].

Lestaurtinib is a potent first-generation type I inhibitor that has been investigated in several clinical trials and indications. It inhibits both FLT3 wildtype and constitutively activated forms at low nanomolar doses. Lestaurtinib has an acceptable toxicity profile, which includes nausea, diarrhea, and (more frequently) infectious complications [43,44]. Importantly, clinical development of lestaurtinib has been discontinued after randomized clinical trials combining lestaurtinib either with first-line or with salvage chemotherapy could not confirm the expected clinical benefit [44,45].

Quizartinib is a highly potent and selective type II inhibitor. It has been investigated in several clinical trials, including the QuantumR study comparing chemotherapy with quizartinib monotherapy in patients with relapsed or refractory AML (R/R AML). Despite a notable clinical benefit, quziartinib is still awaiting approval. Importantly, quizartinib can selectively inhibit ITDs but has no clinically relevant activity against FLT3-TKD mutations. Side effects include QTcF prolongation and “off-target” effect against c-kit, which explains cytopenias accompanying the reduction of bone marrow blasts [46,47].

Crenolanib is a second-generation type I inhibitor targeting both FLT3-ITD and FLT3-TKD mutations. This inhibitor effectively inhibits FLT3 compound mutations (e.g., FLT3-ITD plus D835Y), thereby reducing the risk of secondary resistance. Crenolanib is highly specific without relevant “off-target” activity and shows an acceptable toxicity profile, which includes nausea and vomiting [48,49].

Gilteritinib is a promising second-generation type I inhibitor that has been approved for patients with R/R AML. Patients on gilteritinib showed relevant survival benefit compared to those on chemotherapy regimens in the phase 3 ADMIRAL study. Gilteritinib had an acceptable safety profile, with lower grade diarrhea; liver toxicity; and fatigue among the observed side effects. In contrast to quizartinib, the “off-target” activity of gilteritinib is more favorable, and inhibition of the receptor tyrosine kinase AXL may even reduce the development of secondary resistance [50,51]. Comparison of the ADMIRAL and the QUANTUMR trials revealed a higher rate of grade > 3 febrile neutropenia for patients on gilteritinib (46% versus 31%), while QTc prolongation was more frequent (up to 50%) in patients on quizartinib. Importantly, severe QTc prolongation (grade 3) was rare (3%) [46,50].

## 4. Clinical Activity of FLT3 Inhibitors

Several clinical trials have investigated different FLT3 inhibitors in distinct clinical settings, including monotherapy, combination of either epigenetic approaches or standard chemotherapy regimens (first-line or salvage protocols), and maintenance therapy. In this section, we will focus on early clinical trials focusing on the efficacy of monotherapy regimens (Table 2). Moreover, we will highlight key findings of the RATIFY and SORMAIN trials.

More than a decade ago, midostaurin was evaluated in patients with R/R AML. Monotherapy with this inhibitor demonstrated disappointing results with respect to AML response and survival in this patient cohort [52,53]. In contrast, better results were obtained in clinical trials investigating sorafenib in patients with R/R AML; these trials showed clinically relevant response rates while long-lasting remissions were rare. However, early progression even in patients who achieved a temporary hematological response resulted in short-term survival. Importantly, a survey on patients who underwent ASCT prior to AML relapse demonstrated improved leukemia-free survival compared with those patients who did not receive prior ASCT [40,54].

The RATIFY trial demonstrated improvement of overall survival for all three subgroups of patients with activating FLT3 mutations (FLT3-ITD or FLT3-TKD) by adding midostaurin after intensive induction or consolidation therapy, including maintenance treatment in those patients who did not undergo subsequent ASCT [12]. For this subset of patients the role of maintenance therapy is still a matter of current debate as post-hoc analyses could not demonstrate improvement of disease-free survival [55]. The randomized and placebo-controlled phase 2 SORMAIN trial showed improved relapse-free survival (RFS) in AML patients with FLT3-ITD mutations when sorafenib was applied as maintenance therapy after ASCT. In detail, after 24-months sorafenib treatment RFS was 85% as compared to 53% in the placebo group [41].

The clinical benefit of crenolanib monotherapy was first demonstrated in heavily pretreated patients with R/R AML, including the majority of patients with prior FLT3 inhibitor therapy [49]. In this cohort of 34 evaluable patients, only 12% achieved complete remission with incomplete recovery (CRi). Furthermore, crenolanib has been studied in a small cohort of patients with R/R AML and FLT3-ITD or FLT3-TKD mutations who were not previously treated with any other FLT3 inhibitor. Crenolanib monotherapy resulted in an impressive overall response rate of 50%, including 7/18 patients (39%) achieving complete remission (CRc, composite CR) [49,56]. Larger number of R/R AML patients were included in phase 2/3 studies investigating the second-generation FLT3 inhibitors quizartinib and gilteritinib. Here, quizartinib monotherapy resulted in an overall response rate of about 66%. The subsequent randomized phase 3 QuantumR study demonstrated an excellent overall response rate, with 48% of patients achieving CRc and an overall survival of 6 months for quizartinib treated patients. So far, quizartinib is still awaiting approval for the treatment of patients with R/R AML [46,47]. In contrast, Gilteritinib has recently been approved for the treatment of patients with FLT3-mutated R/R AML. Approval was based on data obtained within the phase 3 ADMIRAL study; here, the authors demonstrated a CRc rate of 54% and an overall survival of 9 months when patients were treated with gilteritinib only. These data were consistent with results of the previously published phase 1/2 clinical trials [50,57].

## 5. Molecular Mechanisms of Resistance

Primary or secondary mechanisms of resistance may affect the clinical response during FLT3 inhibitor therapy.

### 5.1. Primary Resistance

Primary resistance may arise from various cellular mechanisms, including specific FLT3-TKD mutations (either single TKD mutations or compound mutations within the FLT3-ITD allele), mutations in genes other than FLT3, activation of alternative signaling pathways in leukemic cells or the bone marrow niche, and availability of FLT3-TKI.

**Figure 1:**
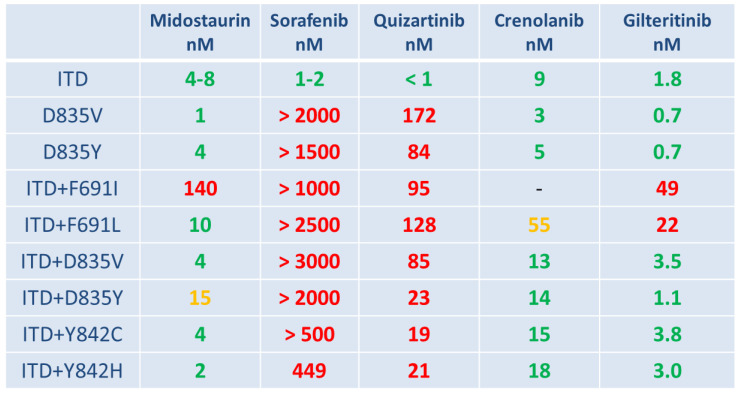
A graphical overview of in vitro sensitivity versus the resistance of FLT3 inhibitors (green—sensitive; orange—intermediate; red—resistant) towards distinct subtypes of activating FLT3 mutations, including compound mutations of FLT3 (ITD plus TKD) [61,62,63,64,65,66,67,68,69].

The presence of a single FLT3-TKD mutation itself can mediate primary compound-specific resistance to certain FLT3 inhibitors. A wide range of FLT3-TKD mutations has been reported to mediate primary resistance against the second-generation inhibitor quizartinib [39]. In particular, the co-occurrence of FLT3-ITD and FLT3-TKD mutations within the same subclone of an individual patient with AML (1–2% of patients at diagnosis) may confer primary resistance to several FLT3 inhibitors (Figure 1). Moreover, patients harboring such compound mutations of FLT3 show lower sensitivity to cytotoxic agents due to increased expression of the anti-apoptotic protein Bcl-x(L) and an impaired cell cycle regulation caused by overexpression of RAD51 [58]. Given the large diversity of FLT3-ITD mutations, the location and amino acid sequence of ITD can also contribute to primary resistance by altering protein conformation that leads to the activation of alternative downstream signaling pathways. The FLT3-ITD variant FLT3-ITD627E confers primary resistance to midostaurin and is characterized by consecutive and inhibitor-independent overexpression of the anti-apoptotic protein MCL-1 [59]. Furthermore, differential sensitivities of FLT3-ITD variants to various kinase inhibitors may also be explained in part by dysregulation of relevant gene-expression programs [60].

Additionally, the bone marrow stroma can contribute to the development of resistance against FLT3 inhibitors via different mechanisms. FLT3-ITD-expressing leukemic stem cells (LSC) are protected within the bone marrow niche. FLT3-mutated leukemic cells are able to reprogram the bone marrow compartment through exosomes, which may lead to suppression of normal hematopoiesis while promoting leukemia (stem-) cell proliferation [70,71]. Second, the metabolism of FLT3 inhibitors by cytochrome P450 3A4 (CYP3A4) is significantly affected by bone-marrow-stromal-cell-expressing cytochromes. In detail, high CYP3A4 expression in the bone marrow microenvironment can prevent effective dose levels in the niche, thus contributing to pharmacological TKI resistance, independent of pharmacologic interactions [72]. Finally, high expression of the chemokine receptor type 4 (CXCR-4) has been reported in FLT3-ITD-positive primary AML cells. Thus, the interaction between FLT3-ITD-positive leukemia cells and bone marrow stroma via the stromal cell-derived factor-1 (SDF1)-CXCR-4 axis has the potential to enhance FLT3 inhibitor resistance [73].

Finally, resistance to FLT3 inhibitors can also be mediated by additional mutations that are not related to FLT3. Pre-existing mutations in the CCND3 gene (encoding cyclin D3) have been described in patients with FLT3-ITD-positive AML who did not respond to the FLT3 inhibitor PLX3397 in an early clinical trial [74].

### 5.2. Secondary Resistance

To understand secondary mechanisms underlying FLT3 resistance, it is important to distinguish between molecular changes within the FLT3-ITD allele (also known as “on-target resistance”) and aberrant signaling that mediates constitutive activation of non-FLT3-dependent oncogenic pathways (“off-target resistance”). Several in vitro models have been used to investigate potential mechanisms of resistance towards different FLT3-TKI. In this section, we will focus primarily on molecular mechanisms of resistance that have been identified in patient samples derived from clinical trials.

The first resistance-mediating mutation was detected in vivo upon midostaurin monotherapy. Molecular analyses in serial samples derived from a patient with R/R AML revealed that a single amino acid substitution at (N676K) within the FLT3 kinase domain conferred resistance to clinically relevant midostaurin trough levels [75]. Following this initial publication, additional point mutations within FLT3 have been shown to mediate secondary resistance against sorafenib. Patient samples from two clinical trials showed resistance-mediating mutations either at the gatekeeper residue (F691) or at codon 835 of the activation loop [54,76]. Molecular analyses revealed A848P mutation as the cause for secondary resistance to sunitinib and sorafenib while preserving sensitivity to midostaurin [77]. Furthermore, several mutations causing resistance against quizartinib, crenolanib, and gilteritinib have been identified.

Quizartinib specifically targets FLT3-ITDs without affecting FLT3-TKD mutations at clinically relevant concentrations. Therefore, secondary resistance of FLT3-ITD cells against quizartinib is mainly reflected by the acquisition of additional point mutations either at the activation loop residue D835 or the gatekeeper residue of the kinase domain (F691) [63]. Targeted sequencing of single cells derived from patients with R/R AML showed polyclonal blast populations harboring several subclones with compound mutations (ITD plus distinct D835 mutations—D835V, Y or F) as well as FLT3-ITD negative subclones with newly acquired FLT3-TKD mutations. These findings indicate that mechanisms of acquired FLT3 inhibitor resistance may not be mutually exclusive, and suggest that the evolution of relevant subclones should therefore be assessed in individual patients at the time of relapse [78].

Crenolanib and gilteritinib represent type I FLT3 inhibitors and show high activity against FLT3-ITD and FLT3-TKD mutations. In contrast to quizartinib, secondary resistance against crenolanib and gilteritinib is rarely caused by FLT3-TKD2 point mutations. For this reason, TET2 and IDH1 mutations have been described predominantly in clones harboring activating FLT3 mutations. In contrast, NRAS and IDH2 mutations were detected in FLT3-independent subclones. Importantly, the co-occurrence of a FLT3 gatekeeper (F691) mutation was detected in only two patients with prior exposure to quizartinib. Development of activation loop mutations could not be identified upon crenolanib treatment [79]. This is consistent with genomic data from AML specimens obtained in clinical trials for gilteritinib treatment. In these trials, 13/41 (32%) patients developed secondary NRAS mutations (3/13 KRAS mutations; 2/13 NRAS and KRAS mutations). F691L resistance-mediating mutations were detected in 5/41 (12%) patients [80]. Recently, preliminary reports have described co-existing or acquired mutations of Janus kinases (JAK1, JAK2, or JAK3) that may confer clinical resistance to sorafenib, midostaurin, or quizartinib in about 4% of patients with FLT3-ITD mutations [81].

Clonal evolution during FLT3 inhibitor treatment may lead to the acquisition of additional oncogenic mutations (e.g., RAS) in FLT3-ITD-positive subclones [80]. The loss of FLT3-ITD mutation at AML relapse has been described by several groups suggesting “off-target” mechanisms of resistance [82,83]. The overexpression of oncogenic kinases (e.g., PIM-2) has been described as a putative mechanism for the development of clinical resistance against sorafenib. Similarly, overexpression of the receptor tyrosine kinase AXL can substantially contribute to FLT3 inhibitor resistance by activation of constitutive STAT5 signaling. As indicated above, the ability of gilteritinib to inhibit AXL downstream signaling may explain improved clinical response and reduced development of resistance [50,84,85]. Finally, sorafenib-resistant FLT3-ITD-positive cell lines and blood samples from AML patients showed aberrant expression of signaling molecules of the PI3K/mTOR pathway, which has been linked to the development of drug resistance [86,87]. Recently, selection of BCR-ABL1-positive clones has been shown in a case series of AML patients who developed resistance to FLT3 inhibitor treatment. While BCR-ABL1 itself is a rare finding in AML, screening for BCR-ABL1 is clinically feasible and may help to identify and target a potential mechanism of resistance [88].

Taken together, various molecular mechanisms confer resistance to FLT3 inhibitors. Importantly, different mechanisms described above are not mutually exclusive, and therefore, highly relevant for diagnostic strategies and therapeutic considerations.

## 6. Molecular Strategies to Overcome Resistance

Understanding the mechanisms of FLT3-inhibitor resistance is the basis for development of diagnostic strategies and therapeutic approaches. Emergence of the F691I/L gatekeeper mutation may vary depending on the choice of FLT3-TKI. While it confers high-level resistance to midostaurin, sorafenib, quizartinib, and gilteritinib, the multi-kinase inhibitor ponatinib may overcome resistance in vitro [89]. Recently, the novel FLT3 inhibitor FF-10101 has also been demonstrated to overcome resistance in FLT3-ITD-F691L-expressing cell lines [90].

Aberrant expression PI3K/mTOR signaling pathway members may not only contribute to FLT3 inhibitor resistance but also suggest therapeutic inhibition of mTOR itself (e.g., with rapamycin). PI3K/mTOR signaling is required to induce apoptosis of FLT3-ITD-expressing AML cells treated with valproic acid and all-trans retinoic acid [87,91]. Additionally, the efficacy and safety of combining epigenetic approaches (e.g., azacitidine) with FLT3 inhibitors (e.g., sorafenib) has been investigated in pre-clinical and early clinical trials. Favorable response rates and good tolerability could be documented in older patients (>60 years) with relapsed FLT3-ITD-AML. Similar results were obtained when combining quizartinib with azacitidine or low dose cytarabine [92,93,94].

The co-occurrence of IDH mutations in FLT3-ITD-subclones (e.g., IDH1) or in non-ITD AML cells (e.g., IDH2) upon giltertinib treatment provides a rationale for the use of IDH-inhibitors either as monotherapy (e.g., enasidenib) or combination therapy (e.g., ivosidenib). This would be of special interest in case of co-occurring RAS mutations in FLT3-independent cells and specifically for the emergence of resistance-mediating subclones harboring NRAS and/or KRAS mutations. Similarly, targeting the MEK/ERK pathway (e.g., with trametinib) or the anti-apoptotic machinery (e.g., with BH3 mimetics) may also represent promising combinatorial approaches to overcome resistance [79,95].

Maturation of FLT3-ITD has been associated with distinct glycosylation-dependent localization or activation of downstream oncogenic signaling pathways. Pharmacological inhibition of FLT3-ITD glycosylation is a potential therapeutic strategy to overcome resistance to FLT3 inhibitors. Fluvastatin or 2-deoxy-d-glucose-mediated inhibition of FLT3-ITD glycosylation results in the retention of the mutated receptor in the endoplasmic reticulum and constitutive activation of the downstream STAT5-PIM-axis [30,96,97]. Furthermore, 2-deoxy-d-glucose may sensitize AML cells to BCL-2 antagonists by affecting the expression of MCL-1 [97]. Constitutive activation of the STAT5-PIM-axis by FLT3-ITD contributes to oncogenic transformation of AML cells and may be involved in conferring resistance to FLT3 inhibitors. Therefore, targeting of STAT5 or PIM isoforms may also represent a combination strategy for overcoming FLT3-TKI resistance [23,98,99,100]. PIM-1 not only stabilizes the hypo-glycosylated 130 kDa FLT3-ITD variant but also inhibits its glycosylation. Furthermore, chaperone proteins, such as HSP90, have been shown to protect FLT3-ITD from proteasomal degradation. The use of HSP inhibitors is another potential strategy for overcoming clinical resistance given the high susceptibility of FLT3-ITD-expressing cells to HSP inhibitors [21,32,101]. Recent data also demonstrate the promising approach of pharmacological inhibition of the Menin-MLL (mixed-lineage leukemia 1-) complex in AML cells with both NPM1 mutation and FLT3 mutation. In detail, combined treatment with FLT3 inhibitors was able to induce apoptosis and to enhance differentiation in AML patient samples [102].

**Figure 2:**
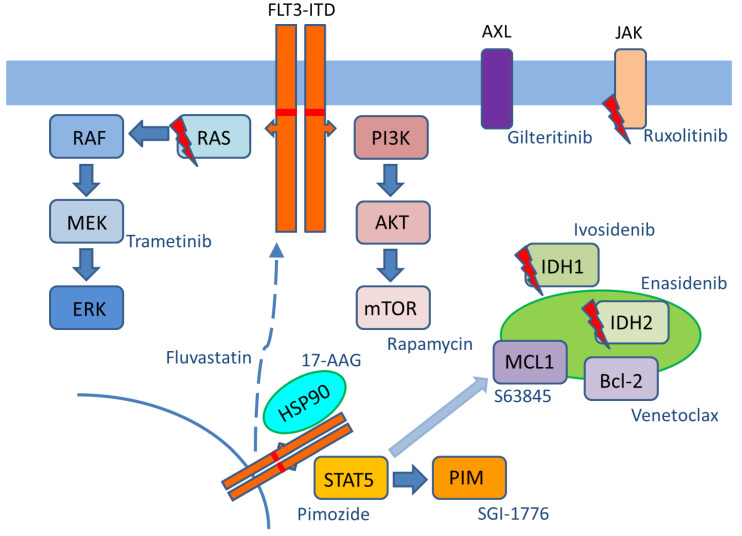
Potential signaling pathways to overcome resistance to FLT3 inhibitors.

Figure 2 gives an overview of signaling pathways that could be targeted to address distinct mechanisms of resistance to FLT3 inhibitors.

Recently, the Bcl-2 inhibitor Venetoclax has been approved in combination with hypomethylating agents for patients inelegible for intensive chemotherapy. It is tempting to speculate whether the combination of venetoclax with FLT3-TKI (and particularly next-generation inhibitors) may be a promising strategy for future clinical development. Recent publications have demonstrated (pre-) clinical synergistic activity of venetoclax in combination with midostaurin, quizartinib, and gilteritinib [103,104]. Table 3 gives an overview of ongoing clinical trials investigating promising strategies of targeted therapy that might contribute to overcoming resistance in AML with activating FLT3 mutations.

Patients with FLT3-ITD mutations receiving an allograft as consolidation therapy have been shown to benefit with improved survival compared to those treated with conventional chemotherapy. Recently, the use of sorafenib in the post-transplant setting has been found to improve leukemia-free survival and the overall survival of patients with FLT3-mutated AML. Mechanisms beyond targeting FLT3-ITD include modulation of graft-versus-leukemia (GvL) responses mediated by IL-15 [105,106,107].

Finally, FLT3-ITD mutations can generate neoepitopes that may be recognized by peptide-specific T-cells with the potential for targeted immunotherapy. Moreover, chimeric antigen receptor (CAR) T-cells directed against FLT3 demonstrate enhanced recognition of FLT3-ITD-positive AML cells after treatment with crenolanib [108,109], a strategy that will be developed in future clinical trials.

## 7. Future Perspectives

Despite the improvement achieved by combining induction chemotherapy with FLT3 inhibitor treatment, FLT3-ITD-positive AML remains a considerable challenge, and allogeneic stem cell transplantation is still a frequent choice for post-induction therapy to reduce the considerable risk of relapse. Development of improved diagnostic approaches by NGS may help to identify relevant AML subclones. Treatment decisions concerning the use of FLT3-TKI in the presence of small FLT3 mutated subclones and detailed molecular characterization of these AML need to be evaluated in future clinical trials.

Resistance to FLT3 inhibitors involves various molecular mechanisms, and improved knowledge of the affected cellular pathways may help to develop personalized treatment strategies. Importantly, advances in diagnostic development are required for comprehensive analysis of leukemic cells, including genomic, transcriptomic, proteomic, and epigenetic approaches.

The type of FLT3 TKI applied in clinical practice may help to anticipate the mechanism of resistance. Additional FLT3 point mutations, acquisition of other mutations (e.g., NRAS, KRAS, IDH1, IDH2), or activation/overexpression of alternative signaling pathways are among the most relevant mechanisms with relevance to guide therapeutic decisions. Development of predictive biomarkers, including intracellular protein activation or expression, may confirm the need for combinatorial approaches (e.g., FLT3 inhibitors and BH3 mimetics) [95,110]. Improved diagnostic characterization of AML beyond FLT3 mutations status and allelic ratio at diagnosis, may help to taylor individualized treatment approaches.

## Figures and Tables

**Table 1:** Selected FMS-like tyrosine kinase 3 (FLT3) inhibitors (FLT3i) evaluated in clinical trials.

FLT3 Inhibitor	Generation and Subtype of FLT3i	Inhibition of FLT3-TKD	“Off Target” Inhibition	Data of Phase 3 Clinical Trial (e.g.,)	FDA Approval for AML
Sorafenib	1st Type II	No	RAF; VEGFR, KIT, PDGFRB	No	No
Midostaurin	1st Type I	Yes	PKC, SYK, SRC, KIT, VEGFR, PDGFR, AKT	Yes [12]	Yes
Lestaurtinib	1st Type I	Yes	JAK2	Yes [44]	No
Quizartinib	2nd Type II	D835Y/V/I/F resistant	KIT, PDGFR	Yes [46]	No
Crenolanib	2nd Type I	Yes	PDGFRB, KIT	No	No
Gilteritinib	2nd Type I	Yes	AXL	Yes [50]	Yes

Abbreviations: FLT3i, FLT3 inhibitor; TKD, tyrosine kinase domain; FDA, Food and Drug Administration.

**Table 2:** Clinical trials containing single agent treatment with FLT3 inhibitors (FLT3i).

FLT3 Inhibitor	AML Setting	Patients (n)	FLT3 Mutation	Phase	Response, n CRc (%) PR (%)	LFS (mo)	OS (mo)	Ref.
Midostaurin	r/r AML AML 1st line	17 2	ITD 18 TKD 1	2	0 (0) 1 (5.2)	n.a.	n.a.	[52]
	r/r AML	35	ITD 26 TKD 9	1	0 (0) 1 (2.9)	n.a.	3.3	[53]
Sorafenib	r/r AML	13	ITD 12 ITD + TKD 1	2	6 (46.2) n.a.	2.4	n.a.	[54]
	r/r AML	65	ITD 65 no TKD	Survey	25 (38)	no ASCT: 4.5 prior ASCT: 6.5	n.a.	[40]
Crenolanib	r/r AML	34	ITD and TKD	2	4 (12%) 1 (3%)	n.a.	4.4	[49]
	r/r AML Cohort A (no prior TKI)	18	ITD 9 TKD 6 ITD + TKD 3	2	7 (39%) 2 (11%)	n.a.	7.8	[56]
Quizartinib	r/r AML	76	ITD 76 no TKD	2	36 (47.4) 14 (18.4)	5.3	22.6	[47]
	r/r AML	245 (allocated to Quizartinib)	ITD 245 no TKD	3	118 (48) 51 (21)	n.a.	6.2	[46]
Gilteritinib	r/r AML	191	ITD 162 TKD 16 ITD + TKD 13	1–2	70 (37) 23 (12)	n.a.	30.0	[57]
	r/r AML	247 (allocated to Gilteritinib)	ITD 215 TKD 21 ITD + TKD 7 Other 4 *	3	134 (54.3) 33 (13.4)	4.4	9.3	[50]

Abbreviations: ASCT, allogeneic stem cell transplantation; CRc—composite complete remission; FLT3i, FLT3 inhibitor; ITD, internal tandem duplication; LFS—Leukemia-free survival, OS—Overall survival TKD, tyrosine kinase domain; PR, partial remission; r/r AML, relapsed or refractory AML; * four patients with unconfirmed FLT3 mutation were assigned to the gilteritinib group.

**Table 3:** Ongoing clinical trials with targeted therapies including patients with activating FLT3 mutations.

ClinicalTrials.gov Identifier	Drug Combination	Targets (Inhibition)	Phase	n	AML
NCT03625505	Venetoclax Gilteritinib	BCL-2 FLT3mut	1b	64	r/r AML
NCT04140487	Venetoclax Gilteritinib Azacitidine	BCL-2 FLT3mut DNA methylation	1/2	42	r/r AML
NCT04336982	CC-9009 Gilteritinib	Cereblon E3 Ligase FLT3-ITD	1/2	66	r/r AML
NCT03735875	Venetoclax Quizartinib	BCL-2 FLT3-ITD	1/2	32	r/r AML
NCT03661307	Venetoclax Quizartinib Decitabine	BCL-2 FLT3-ITD DNA methylation	1/2	52	r/r AML 1L AML unfit
NCT03552029	Milademetan Quizartinib	MDM2 FLT3-ITD	1	156	r/r AML 1L AML unfit
NCT03135054	Omacetaxine Quizartinib	Protein translation FLT3-ITD	2	40	r/r AML 1L AML
NCT03063944	OPB-111077 Venetoclax Decitabine	STAT3 BCL-2 DNA methylation	1	59	r/r AML 1L AML
NCT03132454	Palbociclib Sorafenib	CDK4/6 FLT3-ITD	1	54	r/r AML
NCT03008187	SEL24	Pan-PIM FLT3mut	1/2	45	r/r AML

Abbreviations: 1L, first line; BCL-2, B-cell lymphoma 2; CDK4/6, cyclin dependent kinase 4/6; FLT3mut, activating FLT3 mutation; ITD, internal tandem duplication; MDM2, mouse double minute homolog 2; PIM, serine/threonine-protein kinase; r/r AML, relapsed or refractory AML; STAT3, signal transducer and activator of transcription 3.
